# Engineering *Escherichia coli* for the utilization of ethylene glycol

**DOI:** 10.1186/s12934-021-01509-2

**Published:** 2021-01-22

**Authors:** Aditya Vikram Pandit, Emma Harrison, Radhakrishnan Mahadevan

**Affiliations:** 1 Department of Chemical Engineering and Applied Chemistry, University of Toronto, 200 College Street, Toronto, ON M5S 3E5 Canada; 2 Institute of Biomedical Engineering, University of Toronto, 164 College Street, Toronto, ON M5S 3G9 Canada

**Keywords:** Ethylene glycol, Carbon fixation, Glycolate, Metabolic engineering, Metabolic modeling, Bioprocess optimization, Constraint-based modeling

## Abstract

**Background:**

A considerable challenge in the development of bioprocesses for producing chemicals and fuels has been the high cost of feedstocks relative to oil prices, making it difficult for these processes to compete with their conventional petrochemical counterparts. Hence, in the absence of high oil prices in the near future, there has been a shift in the industry to produce higher value compounds such as fragrances for cosmetics. Yet, there is still a need to address climate change and develop biotechnological approaches for producing large market, lower value chemicals and fuels.

**Results:**

In this work, we study ethylene glycol (EG), a novel feedstock that we believe has promise to address this challenge. We engineer *Escherichia coli* (*E. coli*) to consume EG and examine glycolate production as a case study for chemical production. Using a combination of modeling and experimental studies, we identify oxygen concentration as an important metabolic valve in the assimilation and use of EG as a substrate. Two oxygen-based strategies are thus developed and tested in fed-batch bioreactors. Ultimately, the best glycolate production strategy employed a target respiratory quotient leading to the highest observed fermentation performance. With this strategy, a glycolate titer of 10.4 g/L was reached after 112 h of production time in a fed-batch bioreactor. Correspondingly, a yield of 0.8 g/g from EG and productivity of 0.1 g/L h were measured during the production stage. Our modeling and experimental results clearly suggest that oxygen concentration is an important factor in the assimilation and use of EG as a substrate. Finally, our use of metabolic modeling also sheds light on the intracellular distribution through central metabolism, implicating flux to 2-phosphoglycerate as the primary route for EG assimilation.

**Conclusion:**

Overall, our work suggests that EG could provide a renewable starting material for commercial biosynthesis of fuels and chemicals that may achieve economic parity with petrochemical feedstocks while sequestering carbon dioxide.

## Background

Biological approaches to address climate change and to sequester carbon dioxide (CO_2_) have focused on the development of microbial strains engineered to produce chemicals and fuels derived from renewable sources of sugar [1]. Despite considerable success at engineering these strains at a small scale and the availability of systems biology based approaches for engineering strains, their success at the commercial scale has been hindered by financial limitations, particularly in the face of low oil prices and expensive feedstocks [1, 2]. In response, non-sugar feedstocks have emerged as alternatives. In evaluating alternative, non-sugar substrates, it is important to recognize that many cannot be naturally catabolized by traditional industrial workhorses. Hence, it is necessary to consider the substrate toxicity and biocompatibility, as well as the development of appropriate metabolic pathways for substrate utilization. Furthermore, while certain substrates may be biologically feasible, technical limitations in their own production may render them unusable downstream. While production efficiency and bio-toxicity are more easily assessed, evaluating the feasibility of a new substrate for bio-based chemical production is complicated by how its utilization is linked to the highly interconnected metabolic network. Indeed, refactoring large metabolic pathways in heterologous hosts has proven challenging in the past [3]. One method that may help to explain why a new substrate performs poorly examines the metabolic pathway that supports a substrate for chemical production in relation to the cell’s entire native metabolism [4].

In an earlier study [4], we characterized this relationship by calculating the interactions between two competing objectives of cellular systems; growth and chemical production. The theory laid out how the underlying network structure controls whether chemical production is independent of growth. That relationship was captured by the orthogonality metric which is evaluated by a mathematical framework using elementary flux modes (EFMs) to measure the interconnectedness of the cell system and the desired objectives [5]. We found that the organization of ideal metabolic structures, designed to minimize cell-wide interactions, had a characteristic branched topology. This type of orthogonal structure could be exploited for two-stage fermentation, as it lends itself to the design of metabolic valves for dynamic control [6, 7]. Dynamic control is a strategy employed to increase control over chemical production, often through the temporal segregation of bioproduction from cellular growth [6]. Because of their characteristic branched topology, highly orthogonal pathways often have a key enzymatic step, or metabolic valve, which can be used to control the division of flux for cell growth and chemical production. Various strategies can be used to exert control over these metabolic valves, such as process conditions (pH, temperature, oxygen) or the chemical stimuli of genetic circuits (quorum sensing, inducers, internal metabolite concentration) [6–10]. It seems natural then, that the design of orthogonal pathways, metabolic valves and dynamic control strategies would go hand-in-hand, particularly for the design of two-stage fermentations.

Another important finding from our earlier study [4] was that glucose, while a common substrate for industrial fermentation, is not ideally suited for chemical production objectives due to the significant overlap between the pathways for biomass synthesis and chemical production. Instead, substrate selection should be based on the chemical targeted for production. Among the various substrates and products that we evaluated, we identified that ethylene glycol (EG) was a highly promising substrate for orthogonal production of a variety of chemicals because it minimized the interactions between biomass and chemical producing pathways. Today, EG is produced primarily by the petrochemical industry from ethylene, however, renewable alternatives are currently in the early stages of development [11, 12]. In particular, EG can be produced from the electrochemical conversion of CO_2_ [13, 14], from the chemocatalytic conversion of cellulosic materials and glycerol (a common waste in industrial biofuel and soap production) [11, 12], as well as from the depolymerization of poly(ethylene terephthalate) (PET) plastic (an abundant waste material) to its monomers [15–18]. Thus, though unconventional as a feedstock, EG could serve as a sustainable and/or renewable replacement for glucose in the modern bioprocess.

Though not commonly reported in metabolic engineering applications, there are two main types of naturally existing pathways that allow microorganisms to consume EG as a carbon source [19–22]. The first pathway utilizes a diol-dehydratase resulting in the dehydration of EG to acetaldehyde. Acetaldehyde is then activated to acetyl-CoA by an acetaldehyde dehydrogenase enzyme, which provides the cell with the key pre-cursor metabolite to support growth via the tricarboxylic acid (TCA) cycle and gluconeogenic pathways. This pathway is most commonly found in some *Clostridium* species and a few other anaerobic organisms owing to the oxygen sensitivity of the diol-dehydratase [20, 22]. In the second pathway, EG is successively oxidized using nicotinamide cofactors and oxygen to produce glyoxylate [19, 21]. Glyoxylate, which is a gluconeogenic carbon substrate, can then be used as the growth metabolite as it enters lower glycolysis at the 2-phosphoglycerate node as well as the TCA cycle via the glyoxylate shunt. This oxidative pathway has been shown to exist in a variety of different bacteria [21].

Wildtype *Escherichia coli *(*E. coli*) MG1655 cannot naturally grow on or degrade EG. However, it is possible to select for a strain that does, and to our knowledge, only one study has ever reported EG utilization by *E. coli*. [23]. That strain was selected from derivatives of propylene glycol utilizing mutants. Researchers identified increased activities of propanediol oxidoreductase, glycolaldehyde dehydrogenase and glycolate oxidase as the necessary components required for its assimilation. More generally, a survey of the literature shows that enzyme promiscuity is an essential element of the utilization of alcohols [24, 25]. In this specific case, enzymes regarded as being essential for propanediol or even glycerol utilization across many organisms have shown activity on EG and are regarded as the key methods for degradation, irrespective of the dehydratase route or the oxidative route via glyoxylate [19–21]. Hence, in this study, EG assimilation was engineered in *E. coli* by overexpressing two genes: *fucO* (encoding propanediol oxidoreductase) and *aldA* (encoding glycolaldehyde dehydrogenase). This synthetic pathway is similar to the second natural EG utilization pathway previously introduced: EG is sequentially oxidized to glyoxylate thereby providing a gluconeogenic carbon substrate for growth. More specifically, the promiscuous activity of propanediol oxidoreductase converts EG to glycolaldehyde, which is subsequently converted to glycolate by glycolaldehyde dehydrogenase. The native glycolate oxidase then transforms glycolate to glyoxylate to support cell growth and maintenance.

Motivated by the prospect of utilizing EG as a renewable and alternative feedstock, we sought to compare EG with more conventional feedstocks for the production of select chemicals of industrial significance. In particular, formate, glucose, and xylose were selected as the comparative feedstocks, while succinate, ethanol, glycolate and 2,3-butanediol, were selected as the products of interest. Formate was selected as it is another well-studied, non-sugar feedstock that can be produced via electrochemical CO_2_ reduction (eCO_2_R), and it has already been successfully employed within biological systems [26–30]. Meanwhile, glucose and xylose were selected as typical renewable sugar feedstocks, considering that they comprise the largest fraction of sugars in lignocellulosic biomass [31]. The four products of interest were selected as they are well-known bioproduction targets that have industrial significance [1, 32–34].

In this study, we began by comparatively evaluating EG as a feedstock by measuring the orthogonality of each substrate-product combination, using select bioconversion pathways. Consistent with our previous evaluation [4], EG demonstrated the greatest orthogonality score for all four products considered. For the products investigated, it was determined that the EG-glycolate combination scored the highest based on this metric. Thus, as a case study we engineered and characterized *E. coli* as a biocatalyst capable of growth and glycolate production, using EG. This case study attempts to validate our orthogonal approach for chemical production, relating the network topology and two-stage fermentation.

Glycolate is an alpha-hydroxy acid used in the synthesis of a variety of different plastics and polymers, cosmetics and industrial detergents [32, 33, 35, 36]. Conventional approaches to produce glycolate in *E. coli* have focused on using glucose and/or xylose as a substrate, and typically implement genetic strategies that couple production to growth [31–33, 37–41]. Theoretical yields have been dependent on both the substrate selected as well as the biosynthetic pathway used for production. Examples of glycolate production from glucose in literature have primarily been demonstrated by the activation of the glyoxylate shunt [32, 33, 40, 41], while glycolate production using xylose has been demonstrated by the use of a synthetic pathway for xylose assimilation in *E*. *coli* [31, 37, 38]. More recently, a novel synthetic pathway (named “glycoptimus”) was also designed and constructed in *E. coli* and is predicted to reach molar yields of 2.5 and 3, on xylose and glucose, respectively [39]. However, while the pathway has been shown to be functional in *E. coli*, it has not yet reached the model-predicted yields in vivo. In the identified studies, the highest glycolate titer in *E. coli* is reported to be 65.5 g/L with a corresponding yield of 0.765 g/g*,* using glucose as the substrate [41]. In organisms other than *E. coli*, the highest reported glycolate titer is 110.5 g/L with a corresponding yield of 94.4%, using EG as the substrate. This feat was achieved by Hua and colleagues in *Gluconobacter oxydans* using an integrated production, separation and purification technology [36]. To our knowledge, only five studies have examined EG conversion to glycolate as a biotransformation, none of which were in *E. coli* [36, 42–45].

**Fig. 1:**
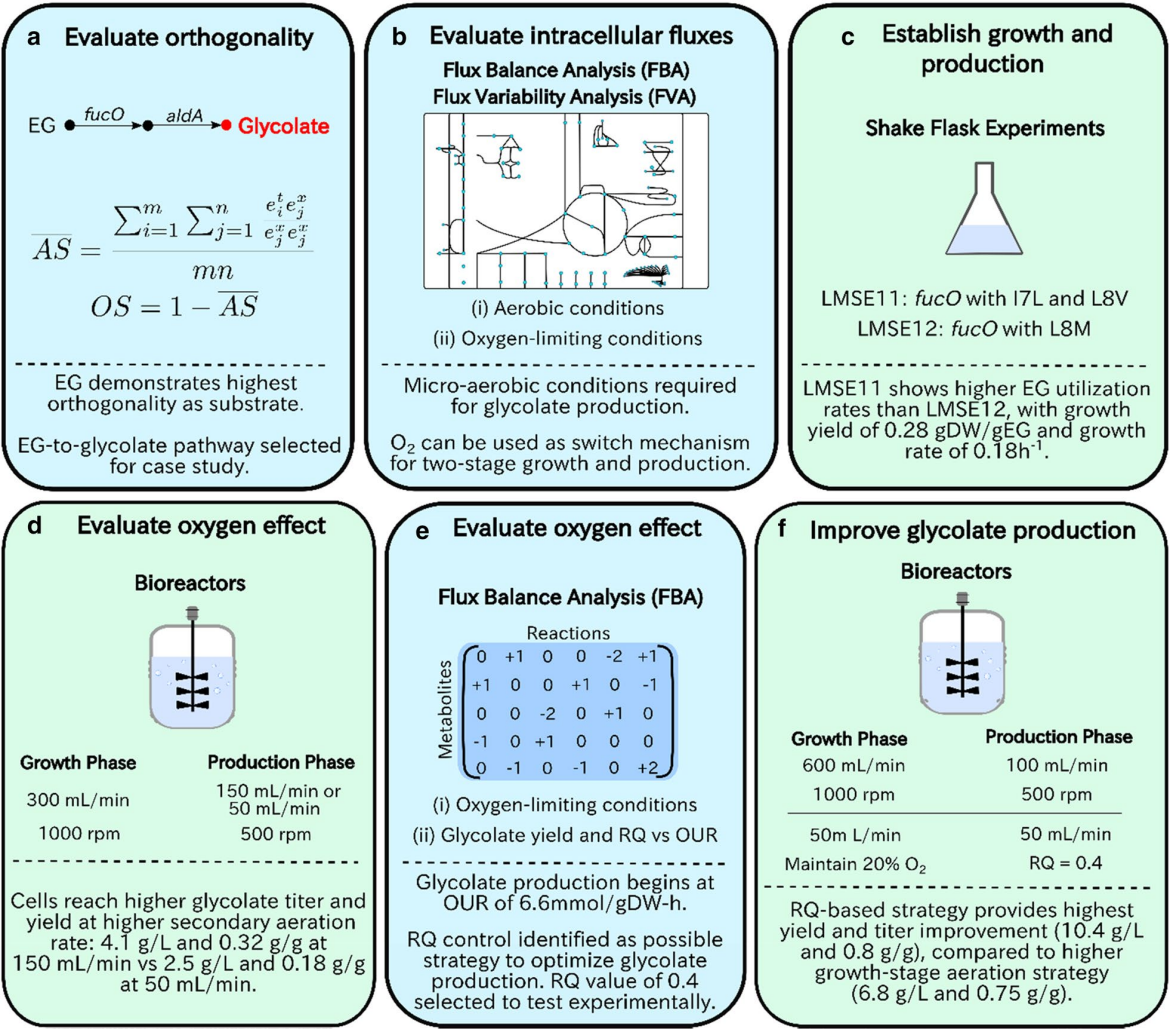
Summary of investigations performed in this study and the key results. **a** The orthogonality (OS) for various substrate-product pairs were evaluated. The most orthogonal pair [ethylene glycol (EG) to glycolate], was selected to demonstrate the use of orthogonality as a metric to establish successful production systems. **b** A stoichiometric metabolic model was built to characterize the production system and its metabolic behaviour using flux balance analysis (FBA) and flux variability analysis (FVA). Both aerobic and oxygen-limiting conditions were used to investigate the effect of oxygen level on growth and production. **c** Two strains employing the selected pathway, each with different enzyme mutants, were then tested in shake flasks to confirm that the pathway could indeed sustain cell growth and glycolate production. **d** The best performing strain (LMSE11) was then tested in bioreactors, using a two-stage growth/production system. As per the identified metabolic valve (glycolate oxidase) and FBA results, a decrease in oxygen level was predicted to switch the system from growth to production. Thus, two secondary air flow rates were tested to evaluate the effect of oxygen level on the production stage. **e** Using data collected from the shake flask and bioreactor experiments, metabolic modeling was used to further characterize the production system and its response to oxygen level. **f** Finally, insights gained from modeling and earlier experiments were used to inform the design and testing of two strategies to improve glycolate production in the bioreactors. Colours indicate the type of analysis performed: blue for computational and green for experimental. Key results are reported below the dotted lines

In this work, we used a combination of computational and experimental investigations to thoroughly characterize the metabolism and growth physiology of *E. coli* growing on EG (Fig. 1). First, we used orthogonality to demonstrate EG’s potential as a substrate and selected the EG to glycolate production pathway as a case study. Next, we characterized the glycolate production system using flux balance analysis (FBA), and showed that the selected pathway supported cell growth through shake flask experiments. Subsequently, two sets of fed-batch growth experiments were performed to optimize growth and production, as well as the use of oxygen as a metabolic valve. Findings from the first growth experiment were combined with a computational study on the effect of oxygen to design strategies for the second set of growth experiments and thus improve pathway performance. Overall, we find that EG has the potential to replace glucose in industrial bioprocesses, particularly in applications where renewable EG can be easily sourced or produced. Further, we demonstrate that computational tools can successfully inform the design and optimization of production systems.

## Results

### Ethylene glycol is a promising substrate

In an earlier study, we identified orthogonality as a metric to assess and design efficient metabolic networks for the production of chemicals [4]. That study defined orthogonality as a quantitative measure of the interconnectedness between pathways that produce a target chemical and biomass. Since then, this principle has been demonstrated in a separate study [7]. More specifically, the orthogonality metric is a mathematical measure of the set of interactions that each substrate assimilation pathway has to the cell components outside their pathways. Hence, it implicitly measures the biological complexity one might expect to ensure that the biomolecular machinery of that pathway can concurrently function within the cell’s natural metabolism to support biological and chemical production objectives. It also allows for metabolic constraints such as redox and ATP to be accounted for. The principal focus of that earlier work was to examine how metabolic pathway organization influences chemical production. Here, orthogonality is used, in addition to yield, as a metric to evaluate the compatibility of specific substrate and product pairs.

We began by first evaluating EG as a feedstock through comparison with three other conventional feed materials: formate, glucose and xylose. Formate was selected as it is a well-developed electrochemical product and has been identified as a good potential feedstock for bioprocesses [26–30]. Glucose and xylose were selected as they are both conventional sugar feedstocks, typically employed in bioprocesses [31]. This feedstock comparison was performed by evaluating the orthogonality of each substrate when used to produce four different chemical products of industrial importance (succinate, ethanol, glycolate, and 2,3-butanediol) [1, 32–34]. While single representative assimilation and conversion pathways were selected for each substrate to product conversion, it should be noted that other pathways exist and continue to be developed which may have different yield and orthogonality scores. For example, xylose utilization can occur via various natural and synthetic pathways, particularly for glycolic acid production [39, 46].

As shown in Table 1, EG consistently demonstrates the highest orthogonality scores for each product. From a yield perspective, EG is predicted to have the highest theoretical yield (g product/g substrate) for both glycolate and 2,3-butanediol, and competitive theoretical yields for succinate and ethanol. Comparatively, while formate demonstrates greater orthogonality than the conventional sugar feedstocks, it scores consistently lower than EG and its predicted yield is notably low across all four products. Analysis of the metabolism of formate shows its lower orthogonality scores, when compared to EG, arises from its low degree of reduction that necessitates flux through the TCA cycle to generate the reducing equivalents required for growth and energy, irrespective of what chemical is produced. Formate’s low degree of reduction is also the reason for its low predicted product yields. Hence, this line of analysis suggests that, from the perspective of ranking non-sugar feedstocks, EG is a superior substrate to formate in *E*. *coli*. Thus, EG was selected for further comparison with the conventional sugar feedstocks, glucose and xylose. Since EG and glycolate demonstrated the highest yield and orthogonality for all substrate-product pairs, this particular pair was selected for further evaluation.

**Table 1:** Yield and orthogonality metrics for chemical production from different substrates

	Succinate		Ethanol		Glycolate		2,3-Butanediol	
	Score	Yield	Score	Yield	Score	Yield	Score	Yield
Ethylene glycol	0.54	0.95	0.61	0.62	0.67	1.22	0.66	0.66
Formate	0.47	0.29	0.5	0.14	0.48	0.33	0.49	0.18
Glucose	0.41	1.12	0.44	0.51	0.41	0.85	0.47	0.50
Xylose	0.36	1.12	0.36	0.51	0.34	1.01	0.4	0.50

The orthogonality scores for various products are shown comparing two substrates that can be generated electrochemically (EG and formate) against conventional sugar substrates (glucose and xylose) assimilated via their natural pathways. Formate has orthogonality scores similar to many sugar consuming pathways, indicating its utilization is relatively complex and interconnected with native growth. Ethylene glycol (EG) exhibits the highest orthogonality scores, and has higher or comparable theoretical yields relative to the other substrates. Yield is given as g of product per 1 g of substrate

**Fig. 2:**
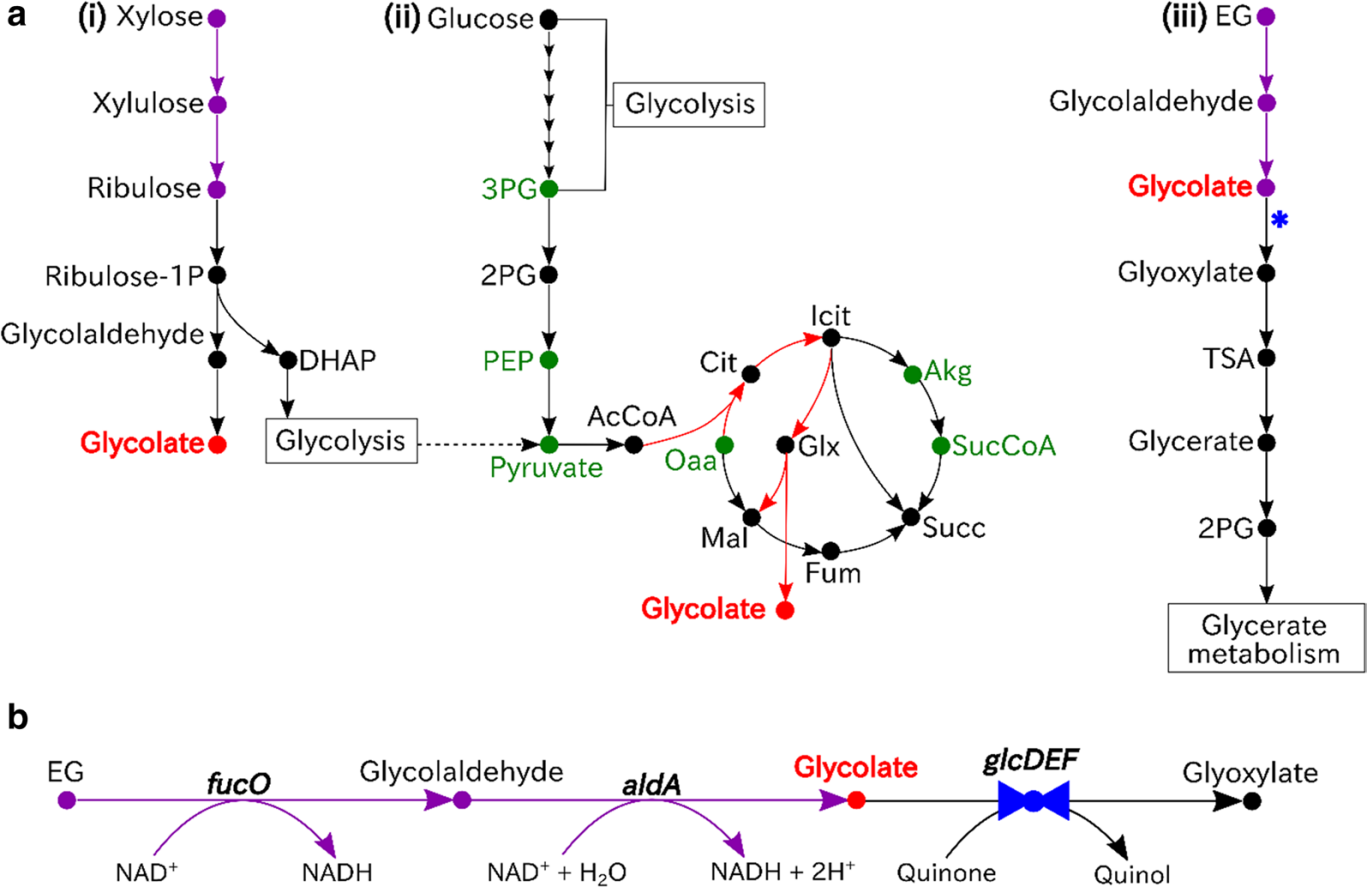
Comparison of glycolate bioproduction pathways from substrates of interest. **a** Glycolate can be bioproduced from a variety of different substrates, including (i) xylose, (ii) glucose and (iii) ethylene glycol (EG). Xylose and glucose are the two most commonly studied substrates; however, glycolate production from these substrates is limited by the interconnectedness of the growth and production pathways, as indicated by their topology and low orthogonality scores. Consequently, efficient production of glycolate from either of these compounds necessitates the coupling of growth and glycolate synthesis. Alternatively, EG assimilation lends itself to a branched topology that permits the decoupling of glycolate synthesis and cell growth (high orthogonality score). **b** Although *Escherichia coli* (*E. coli*) cannot naturally assimilate EG, the conversion of EG to glycolate can be introduced via the overexpression of a mutant propanediol oxidoreductase (encoded by *fucO*) and glycolaldehyde dehydrogenase (encoded by *aldA*). The subsequent conversion of glycolate to glyoxylate provides a branch point that serves as a metabolic valve. Under fully aerobic conditions, glycolate is converted to glyoxylate and channeled to the central metabolism for growth via glycerate metabolism. Alternatively, under oxygen limiting conditions glycolate accumulates. Oxygen can thus be used for dynamic control of this metabolic valve, thereby decoupling the production of glycolate and biomass. Colours denote the following: exogenous steps (purple), the desired product (red), biomass precursors (green), and metabolic valves (blue). For production from glucose (A (iii)), red arrows are also used to specify the product pathway. *DHAP* dihydroxyacetone phosphate, *3PG* 3-phosphoglycerate, *2PG* 2-phosphoglycerate, *PEP* phosphoenolpyruvate, *AcCoA* acetyl-CoA, *Cit* citrate, *Icit* isocitrate, *Akg* alpha-ketoglutarate, *SucCoA* succinyl-CoA, *Succ* succinate, *Fum* fumarate, *Mal* malate, *Oaa* oxaloacetate, *EG* ethylene glycol, *TSA* tatronate semialdehyde

Figure 2a shows the glycolate production pathways selected for xylose, glucose and EG. For the three pathways shown, xylose exhibits the lowest orthogonality score (0.34), owing to glycolate production being highly coupled to biomass synthesis. In this pathway, the biomass precursor, DHAP, and the glycolate precursor, glycolate, are concomitantly produced. Consequently, when using this pathway, it is impossible to separate chemical and biomass production, as one necessitates production of the other. Production from glucose is also highly coupled to biomass synthesis, and exhibits a low orthogonality score (0.41). While this pathway fits partly into an orthogonal criterion for glycolate production, the concomitant production of pyruvate for every mole of glycolate requires the use of the cell’s highly interconnected glyoxylate cycle to reach theoretical yields. The orthogonality score, for this reason, is comparatively smaller than that of EG.

As previously reported in Table 1, the production of glycolate from EG exhibits the highest orthogonality score (0.67). Unlike xylose and glucose, EG is not naturally assimilated by *E. coli*, however it can be engineered to do so through incorporation of the pathway shown in Fig. 2b. As previously noted, *E. coli* naturally possesses 1,2-propanediol oxidoreductase (*fucO*), for which mutants have reportedly shown promiscuous activity with EG [23]. This observation forms the basis of the engineered EG assimilation pathway. Using a suitable propanediol oxidoreductase mutant, EG is first converted to glycolaldehyde and subsequently transformed to glycolate through the action of glycolaldehyde dehydrogenase (*aldA*). From here, glycolate can be accumulated as the desired product, or further converted to support cell growth. In the latter case, glycolate oxidase converts glycolate to glyoxylate to support cell growth via native metabolic pathways. This pathway fits the ideal network architecture of a branched pathway, for which a metabolic valve can be designed and implemented for dynamic control of production and biomass fluxes. More specifically, in the EG-glycolate pathway shown, the glyoxylate oxidase reaction naturally provides a suitable metabolic valve controllable by oxygen levels. Under fully aerobic conditions, glycolate is converted to glyoxylate and channeled to the central metabolism for growth via glycerate metabolism. Alternatively, under oxygen limiting conditions, this reaction can be limited, and the cell can accumulate glycolate.

Ultimately, as evidenced by the orthogonality scores and the pathway topologies for glycolate production, EG is more orthogonal than the traditional substrates investigated, and hence suitable for validating the concept of pathway design based on orthogonality. Thus, we performed a case study for the production of glycolate from EG in *E. coli* MG1655. In this case study, a combination of computational and experimental evaluations were performed to characterize this production pathway, and to explore its implementation within a two-stage fermentation system.

### Modeling growth of *E. coli* using EG

**Fig. 3:**
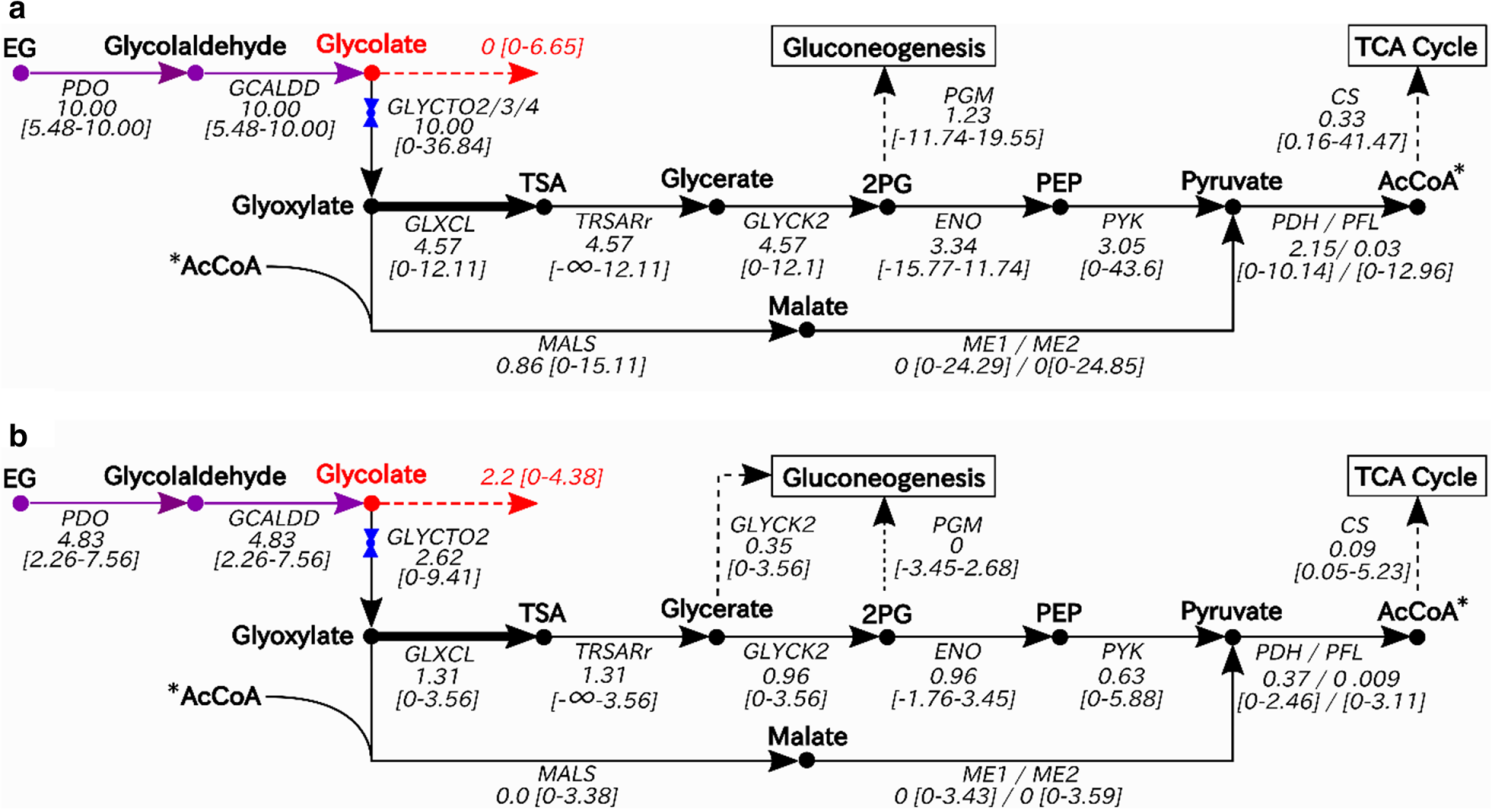
Intracellular flux distribution for EG metabolism under** a** aerobic and** b** micro-aerobic (oxygen-limiting) conditions. Fluxes predicted by flux balance analysis (FBA) are shown under each reaction arrow. Values in the brackets (units: mmol/gDW hr) represent the lower and upper values obtained from the flux variability analysis (FVA). The FVA flux ranges provide an estimation of the error in the reaction fluxes predicted by FBA, for the selected simulation constraints. Enzymes catalyzing each step are indicated under each reaction arrow. Enzyme acronyms are as follows: PDO (1,2-propanediol oxidoreductase), GCALDD (glycolaldehyde dehydrogenase), GLYCTO2 (glycolate oxidase, ubiquinone-dependent), GLXCL (glycolate carboligase), TRSARr (tartronate semialdehyde reductase), GLYCK2 (glycerate kinase), PGM (phosphoglycerate mutase), ENO (enolase), PYK (pyruvate kinase), MALS (malate synthase), ME1/ME2 (malic enzyme, NAD^+^/NADP^+^-dependent), PDH (pyruvate dehydrogenase), PFL (pyruvate formate lyase), and CS (citrate synthase). Compound acronyms are as follows: EG (ethylene glycol), TSA (tartronate semialdehyde), 2PG (2-phosphoglycerate), PEP (phosphoenolpyruvate), AcCoA (acetyl-CoA). Colours denote the following: exogenous steps (purple), the desired product and its export (red), and metabolic valves (blue). Increased line thickness for the glyoxylate to TSA reaction indicates the 2:1 reaction stoichiometry, accounting for the reduction in total flux observed at the glyoxylate node

To gain insight into the expected metabolic behaviour, the intracellular fluxes of the production system were first investigated using flux balance analysis (FBA) and flux variability analysis (FVA). For this analysis, the *E. coli* core model was modified with the addition of the EG assimilation pathway previously shown in Fig. 2a, encompassing the conversion of EG to 2-phosphoglycerate (2PG). Transport and import/export reactions were also added for EG, assuming it crossed the membrane by free diffusion. FBA and FVA were performed under both aerobic and micro-aerobic (oxygen-limiting) conditions, to investigate how oxygen level influences growth and production. Results for the FBA and FVA analyses are summarized in Fig. 3.

Under aerobic conditions (Fig. 3a), FBA predicts a biomass production rate of 0.30 h^−1^ (mass yield of 0.49 gDW/gEG), no glycolate production and no by-product production other than CO_2_ (ie. acetate, ethanol, etc.). Approximately 91% of the glyoxylate flux is channeled towards 2-phosphoglycerate (2PG) and enters lower glycolysis. The remaining glyoxylate is used to generate malate via malate synthase. Of the total carbon (EG) entering the cell, 22% is channeled towards acetyl-CoA and 3.3% enters the TCA cycle. Conversely, about 18% of the total carbon is channeled by gluconeogenic pathways towards upper glycolysis and the pentose phosphate pathways, when accounting for stoichiometry.

Under micro-aerobic conditions, modeling suggests that glycolate production will occur (Fig. 3b). More specifically, the model predicts a biomass production rate of 0.087 h^−1^ and a glycolate export flux of 2.2 mmol/gDW·h. These values correspond to a biomass mass yield of 0.29 and a glycolate mass yield of 0.56. The only by-product observed under micro-aerobic conditions is CO_2_. The FVA values reported for both aerobic and micro-aerobic conditions provide a range of flux values that may be possible. In the case of the tartronate semialdehyde reductase (TRSARr) reaction, the large lower bound flux predicted by FVA indicates that alternate optima exist for this system, for the set of constraints used. Overall, the FBA results suggest that micro-aerobic conditions are required for the secretion of glycolate and that oxygen level could be used as an effective control mechanism for switching from growth to production. In the sections that follow, we attempt to experimentally validate the predicted link between oxygen levels and glycolate production and to use modeling to better understand this relationship. Applying these insights, we then develop and test two strategies to optimize glycolate production in *E. coli,* using EG as the feed material.

### Establishing ethylene glycol utilization by *E. coli*

**Fig. 4:**
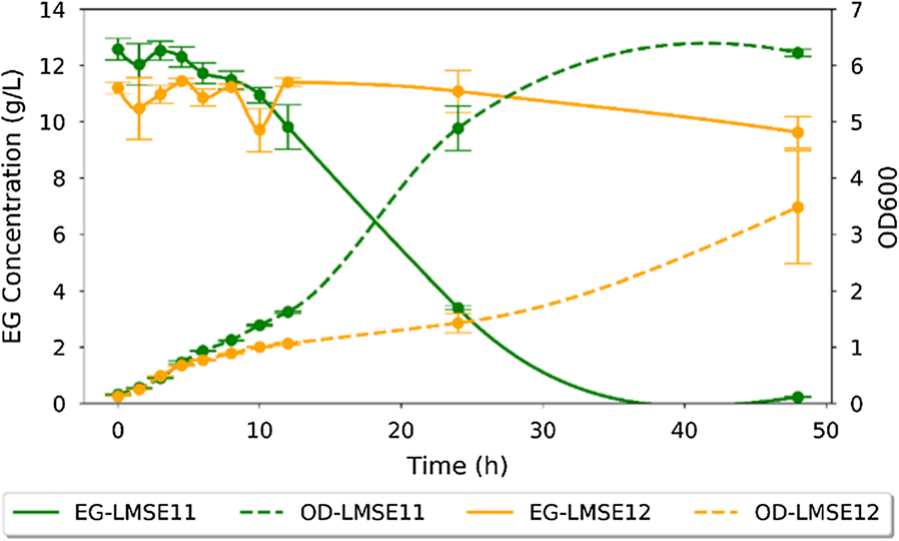
Cell growth and substrate consumption profiles for the two strains constructed in this study. The oxygen variants of *fucO* (strains LMSE11 and LMSE12) showed a marked difference in growth rate and substrate utilization in shake-flask experiments. Ethylene glycol (EG) consumption is shown by the dashed lines and cell growth, as measured by OD600, is depicted by the solid lines. Yellow (light) shows strain LMSE11 while green (dark) shows LMSE12. Error bars indicate standard deviation for triplicate experiments

As previously described and predicted computationally, EG assimilation and conversion to glycolate can be introduced in *E. coli* through the expression of a suitable 1,2-propanediol oxidoreductase mutant (*fucO*), and glycolaldehyde dehydrogenase (*aldA*). Previous studies have shown that Fe^2+^-dependent propanediol oxidoreductases (encoded by *fucO*) can be inactivated by metal-catalyzed oxidation (MCO) and are therefore sensitive to oxygen [47]. Thus, we designed two variants of the pathway, each containing *fucO* mutants reported as being more oxygen-stable. Variant 1 (strain LMSE11) contained *fucO* with mutations I7L and L8V, as reported by earlier mutagenesis studies [47]. Variant 2 (strain LMSE12) contained *fucO* with a single L8M mutation, as it was also suggested to play a role in alleviating metal catalyzed oxidation (MCO) toxicity in propanediol assimilation by *E*. *coli* [48]*.* Both variants had the same ribosome binding site and *trc* promoter upstream of the start codon. As shown in Fig. 4, the fermentation profiles for the two constructed strains were markedly different. LMSE11 completely consumed EG in 47 h while LMSE12 had consumed only ~ 10% of the initial substrate in the same time period with 10 g/L as residual EG. Growth yield for LMSE11 was calculated to be 0.28 gDW/g EG. Comparatively, a theoretical yield of 0.49 gDW/gEG was predicted by flux balance analysis (FBA) using the *E. coli* iAF1260 model (previously described). Thus, the experimental results suggest that the two genes introduced (*fucO* and *aldA*) are sufficient for supporting the conversion of EG to biomass, when combined with *E*. *coli’s* natural biosynthetic pathways. However, the actual yield is less than 60% of the model-predicted yield, thereby suggesting that the pathway may not be operating optimally (i.e. oxygen sensitivity) or that the modeling may need additional constraints. For example, the substrate uptake rate in shake-flasks was determined to be 5 mmol/gDW·h, compared to the 10 mmol/gDW·h assumed previously for modeling. When the modeling is performed instead for an EG uptake flux of 5 mmol/gDW·h, the predicted biomass yield decreases to 0.43 gDW/gEG. Similarly, EG transport was modelled as free diffusion, however if in reality proton symport is required, it may further limit the achievable yield. Oxygen-limiting conditions could also explain a lower than optimal yield, however this is unlikely since analysis of the fermentation media by high-performance liquid chromatography (HPLC) showed the absence of intermediate metabolites (glycolaldehyde and glycolate) and fermentation products, such as acetate or lactate. The experimental growth rate was calculated to be 0.18 h^−1^, corresponding to a 3.85 h doubling time. Since LMSE11 showed higher utilization rates, this variant was used in subsequent experiments.

### Effect of oxygen on two-stage glycolate production in *E. coli*

Having established EG utilization by an engineered strain of *E*. *coli*, we next explored the use of EG as an orthogonal substrate for the production of glycolate within a two-stage fermentation system. As previously described, the branched topology of the EG-glycolate pathway lends itself to the design of a metabolic valve for the separation of cell growth and chemical production. In particular, glycolate oxidase was identified as a potential metabolic valve, for which oxygen level was predicted to be a control mechanism. A higher oxygen level is expected to support biomass growth, while lower oxygen levels are expected to trigger glycolate accumulation. Thus, two reductions in oxygen (air flow rate) were tested to evaluate the effect of oxygen on glycolate production.

**Fig. 5:**
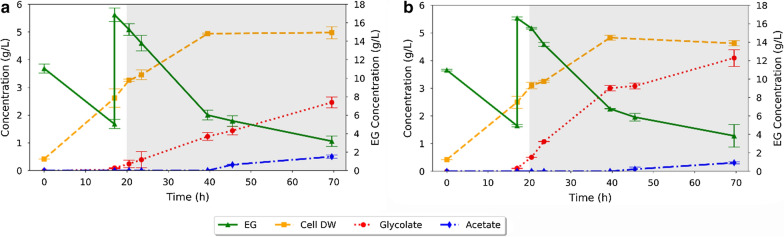
Influence of aeration on glycolate production. To assess the impact of oxygen level in bioreactors, two aeration rates were tested during the production (micro-aerobic) phase of the fermentation. A flow rate of 50 mL/min was tested for the low aeration rate (**a**), while a flow rate of 150 mL/min was tested for the high aeration rate (**b**). Experiments were performed in duplicate. Error bars indicate the range of the measured values

For this evaluation, LMSE11 (variant 1) was grown in bioreactors with minimal media, supplemented with 2 g/L of yeast extract. The bioreactors were inoculated at an initial OD of ~ 0.4 (approximately 0.23 gDW/L), with inoculum prepared as described in the methods. Expression of the EG utilization genes was induced with 1 mM IPTG. During the growth stage, the impeller agitation was set at 1000 rpm, and the reactors were sparged with air to maintain an aeration rate of 300 mL/min (1 v/vm). At 20 h, the aeration was reduced to 150 mL/min (0.5 v/vm) or 50 mL/min (0.16 v/vm), to simulate high and low aeration rates, and the impeller agitation was dropped to 500 rpm (Fig. 5). We observed that cell growth continued until approximately 40 h, reaching approximately 5 gDW/L, at which point cells in both reactors appeared to enter a stationary phase. Production of glycolate started at approximately 20 h and continued until the fermentation was terminated at 70 h. Cells grown at a higher secondary aeration rate (150 mL/min) accumulated more glycolate by the end of the batch (Fig. 5b), reaching a final glycolate titer of 4.1 g/L, compared to 2.5 g/L for the lower secondary aeration rate (50 mL/min) (Fig. 5a). Similarly, the average mass yields for glycolate on EG, as measured during the production phase, were 0.32 g/g and 0.18 g/g, for the high and low secondary aeration rates, respectively. Using FBA to approximate carbon loss from respiration and accounting for cell growth and acetate production, we were able to close the carbon balance at 83% and 88%, respectively. This carbon balance was performed by determining the moles of EG required to supply the carbon in each mole of product (glycolate, acetate and biomass). In the case of biomass, this value was determined using FBA and together these values accounted for the total expected CO_2_ production. These values were then converted to the mass of EG required and compared to the actual mass of EG consumed, leading to the values reported above. Further refinements to the FBA model for *E. coli* growth on EG may close the carbon balance with improved accuracy.

Counter-intuitively, despite propanediol oxidoreductase (*fucO*) being oxygen-sensitive, it was observed that the higher secondary aeration rate (150 mL/min) led to higher glycolate titers (Fig. 5b). This result can be explained by the fact that oxygen is required for the regeneration of NAD^+^, which is a substrate for the EG utilization pathway (see Fig. 2b). Hence, lower oxygen concentrations could lead to reduced flux through this pathway resulting in lower titers. These results suggest that there is a trade-off between the oxygen sensitivity of propanediol oxidoreductase (*fucO*) and the oxygen-dependent regeneration of NAD^+^ required by the pathway. Based on these results, we turned to metabolic modeling to computationally evaluate the effect of oxygen and thus determine a strategy for improved glycolate production in vivo.

### Effect of oxygen on ethylene glycol metabolism in *E. coli*

**Fig. 6:**
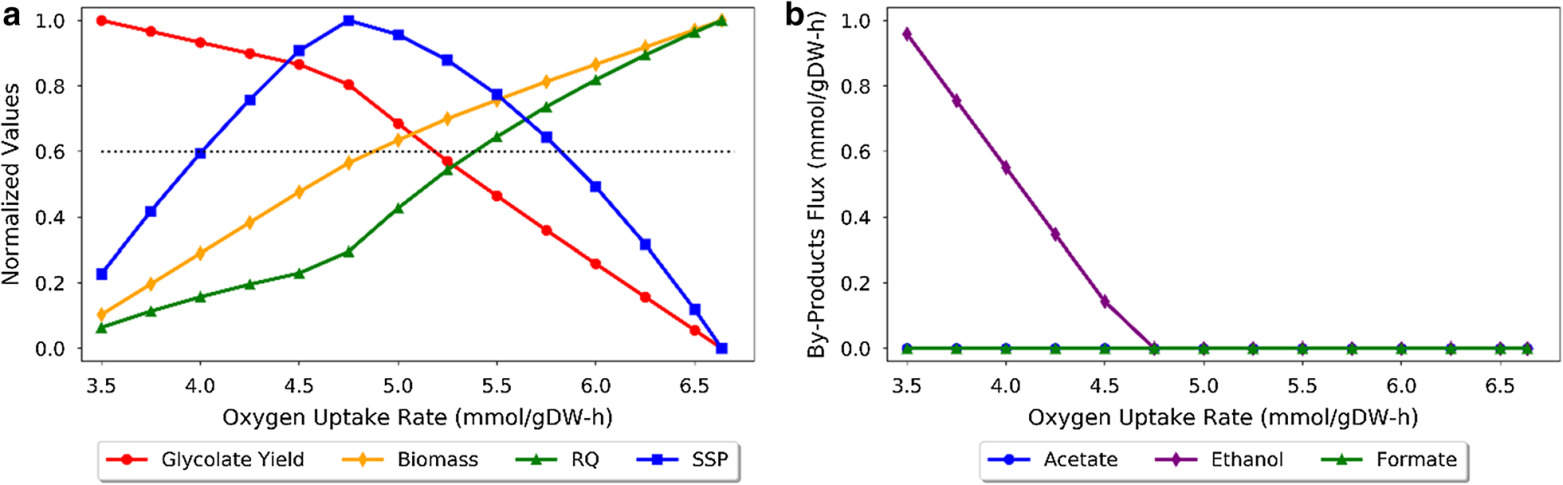
The effect of microaerobic (oxygen-limiting) conditions on glycolate production, as predicted by modeling. **a** Flux balance analysis (FBA) was used to predict the glycolate yield (mol glycolate/mol EG), cell growth rate (gDW/gDW·h), respiratory quotient (RQ, mol CO_2_/mol O_2_) and the substrate specific productivity (SSP, g glycolate/g EG·h) over a range of oxygen uptake rates (OURs, mmol O2/gDW·h). These values were then normalized with respect to their highest values, and are presented here as: Glycolate Yield (red circle), Biomass (yellow diamond), RQ (green triangle) and SSP (blue square). The normalized RQ value corresponding to the selected RQ value of 0.4 is shown by the dotted black line. **b** FBA was also used to explore by-product fluxes (mmol/gDW·h) over the same range of oxygen uptake rates (OURs). The by-products shown are those most commonly produced under fermentative conditions: acetate (blue circle), ethanol (purple diamond) and formate (green triangle). The FBA simulations were performed using biomass as the objective and with the EG flux set to 5 mmol/gDW·h, as measured during the shake flask experiments. The simulations predict that glycolate production begins at the onset of oxygen limitation, at approximately 6.6 mmol/gDW·h of oxygen. At greater OUR values, FBA predicts that there is no glycolate accumulation or by-product production and that the RQ and cell growth plateau, since enough oxygen is available for complete respiration

To refine our strategy for glycolate production, FBA simulations were used to gain further insight into the cell’s metabolic response to changes in oxygen. To characterize the effect of oxygen, the production of the glycolate (yield), biomass production (biomass flux), the cell’s respiratory quotient (RQ; ratio of carbon dioxide emitted to oxygen consumed), and the substrate specific productivity (SSP) were modelled as a function of the oxygen uptake rate (OUR). The formation of by-products was also followed over the same OUR range. The normalized results are shown in Fig. 6, while the raw values prior to normalization are shown in Additional file 1: Figure S1. The modeling results support the use of oxygen as a mechanism to switch from the growth phase to the production phase, within the glycolate production system. As shown in Fig. 6a, glycolate production begins at a limiting oxygen uptake rate (OUR) of approximately 6.6 mmol/gDW·h, and glycolate yield continues to increase as oxygen uptake is further reduced. Contrarily, the biomass flux and RQ both increase with the OUR up to a maximum value. This maximum value depends on the flux of EG, and corresponds to the same limiting OUR (~ 6.6 mmol/gDW·h) at which glycolate production commences. This limiting OUR value changes depending on the EG flux. The modeling also predicts that by-product formation occurs when the OUR drops below 5.25 mmol/gDW·h, and that the type and amount of by-product formation (namely ethanol) depends on the OUR relative to the EG flux.

Since the specific oxygen uptake rate is a function of air intake, this analysis allowed us to implicitly correlate the glycolate yield to the flowrate of air into the reactor. Furthermore, as the RQ and glycolate yield can be correlated via their relationship to the OUR, it should also be possible to optimize glycolate production in real-time by measuring and controlling the RQ throughout a fermentation experiment. Controlling the RQ was thus identified as a possible strategy to improve glycolate production. The selected RQ must balance the glycolate yield, substrate specific productivity and cell growth, to optimize glycolate production. The substrate specific productivity (SSP) is a measure of the moles of product obtained per mole of substrate per time (molP/molS h), and is calculated as the product of the product yield and cell growth rate (Y_PS_·μ). Based on Fig. 6, operating between an RQ value of ~ 0.15–0.4 is most optimal when considering the yield, SSP and cell growth. Therefore, to test whether this approach would increase glycolate production, an RQ value of 0.4 (corresponding to a normalized value of ~ 0.6) was selected to test experimentally.

### Improving glycolate production

**Fig. 7:**
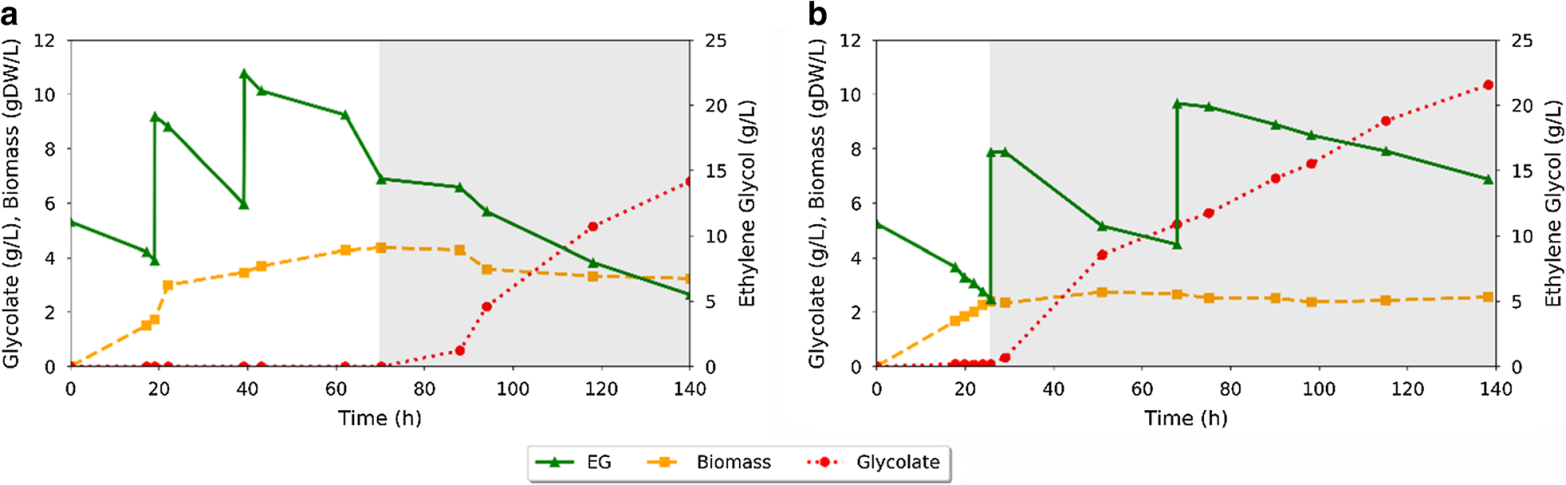
Fermentation profiles for aeration strategies. Two different aeration strategies were tested to improve glycolate production. The growth phase and production phase are separated by grey shading. **a** The first strategy employed a high flow rate of 600 mL/min during the growth phase, and a flow rate of 100 mL/min during the production phase. The final growth phase cell density was approximately 4 gDW/L and the final glycolate titer reached 6.8 g/L. **b** The second strategy used a consistently low flow rate of 50 mL/min during both the growth and production phases, but the impeller speed was reduced during the production phase such that the respiratory quotient (RQ) was approximately 0.4. The average stationary phase cell density was 2.5 gDW/L. Cells were capable of robust glycolate production for well over 100 h in the production phase, reaching a final glycolate titer of 10.4 g/L

Informed by experimental and computational findings, two strategies were tested to increase glycolate production yield and titers. In the first strategy, a higher aeration rate was tested in the growth phase, while in the second strategy the RQ value was used to control the aeration rate in the production phase (as suggested via modeling). For both strategies, bioreactor experiments were performed as previously described, but with modified aeration rates and mixing speeds. The first strategy was tested with the goal of reducing the biomass production phase and increasing the glycolate production phase. To achieve this, the aeration rate during the growth phase was increased to 600 mL/min (2 v/vm) to prevent glycolate accumulation and divert flux towards cell growth. In the second phase, the aeration rate was dropped to 100 mL/min. Results for this strategy are shown in Fig. 7a.

Cell growth continued for approximately 70 h, after which the reactor appeared to reach stationary phase. Production of glycolate began at approximately 70 h and continued until the fermentation was terminated at 140 h (70 h of production). Final glycolate titers reached 6.8 g/L, with an initial production phase biomass concentration of approximately 4 gDW/L, corresponding to an average productivity 0.1 g/L h or approximately 0.32 mmol/gDW·h. The initial yield of glycolate was 0.92 g/g after the first sample was taken, however, the cumulative yield decreased during the course of the production stage with the final overall production yield being 0.75 g/g, or 61% of theoretical (see Table 1). 

In implementing this first strategy, we produced significantly more product at a higher yield; however the cells took much longer to reach an appropriate concentration for the production phase. At 600 mL/min (2 v/vm) during the growth phase, it took almost 70 h to reach a concentration of 4 gDW/L, while comparatively the previously tested 300 mL/min (1 v/vm) produced the same cell concentration within 30 h. We hypothesize that it took longer to reach a higher OD due to the oxygen sensitivity of propanediol oxidoreductase (*fucO*). Even using the more oxygen-stable mutant (strain LMSE11), the increased dissolved oxygen levels and faster oxygen mass transfer rates likely caused oxygen toxicity in the cells due to the inactivation of propanediol oxidoreductase by MCO. This likely placed a high metabolic burden on the cell stemming from high protein demand without a sufficient means to utilize EG as a carbon source. Considering all of the bioreactor experiments up to this point, it seems that higher aeration rates lead to higher glycolate titers and yield, yet also retard cell growth when too high.

From a process perspective, meeting the oxygen demand in large-scale aerobic bioreactors often requires significant energy input, making it a major operating cost [49]. Thus, if possible, it is desirable to operate reactors at a lower air flow rate. With this in mind, the second strategy we employed sought to produce glycolate at a high titer but at a lower aeration rate. Hence, cells were cultivated under a constant low aeration rate of 50 mL/min (0.17 v/vm), but at a variable impeller speed. In the growth stage, the impeller speed was controlled such that the oxygen level remained above 20% (up to a maximum of 1000 rpm). In the production phase, the impeller speed was decreased to reduce oxygen transfer. As mentioned previously, our FBA simulations showed a correlation between the RQ and the glycolate yield, and we stipulated that this relationship could be used to optimize glycolate production. From this computational analysis, we determined that an RQ value of ~ 0.4 was within the optimal operating region. Therefore, the production stage impeller speed was decreased until the RQ, as measured by the online-mass spectrometer, reached the selected value of ~ 0.4.

The RQ was first measured at the start of the production stage at 26 h, and was measured at multiple intervals over the remainder of the 140-h fermentation. Overall, the average RQ value during this time was measured as 0.37. The results of this experiment are shown in Fig. 7b. Using this RQ-based strategy, the growth stage was reduced to 26 h, with a final cell concentration of approximately 2.4 g/L at the end of the growth phase. Glycolate production began at approximately 28 h and continued until the fermentation was terminated at 140 h. During the 112 h of production time, 10.4 g/L of glycolate was produced with overall yield of 0.8 g/g from EG. The productivity was determined to be 0.1 g/L h, making it comparable to the productivity measured in the first strategy (high aeration rate). These experimental results suggest that the RQ can indeed be used as an effective control variable, as predicted by FBA. However, while FBA simulations predicted that an RQ value of ~ 0.4 would reach a glycolate molar yield of ~ 0.4 mol/mol, experimentally this strategy led to a molar yield of 0.66 mol/mol. These results suggest that while the FBA simulations were useful in identifying a control strategy to improve glycolate production from EG, further optimization of model parameters is required to accurately predict the physiological response to environmental conditions. Therefore, the substrate uptake rate along with the ATP maintenance parameters are important parameters that may need further investigation [50, 51]. This is further discussed in Additional file 1: Figure S1.

## Discussion

Conventional approaches to the bio-based production of chemicals rely heavily on sugar-based feedstocks, such as glucose and xylose. Yet, microorganisms tend to be very diverse in their ability to metabolize different carbon sources. In this work we proposed and examined the use of EG as a non-sugar alternative to support growth and chemical production in bioprocesses. One of the greatest motivations in studying EG as a substrate stems from its ability to be derived from CO_2_, either through electrochemical reduction or other conversion technologies [52, 53]. Hence, its consideration as a feedstock that can potentially sequester carbon is akin to studies examining syngas or formate utilization.

By evaluating the orthogonality of selected substrate-product pairs, the conversion of EG to glycolate was identified as a good candidate to assess EG utilization in the context of biochemical production. From the results obtained in this study, we conclude that EG is a suitable platform for growth and highly efficient for producing glycolate. More generally we also believe that with further metabolic engineering, EG could be used to produce alcohols and other organic acids that are typically produced during fermentative metabolism. This capability, we believe, can have an impact in industrial biotechnology.

Our consideration for EG as a substrate was driven primarily by challenges related to the utilization of sugar-based substrates in *E*. *coli*. As we described earlier, the amount of interactions between growth and production pathways affects the level of production that can be achieved. These interactions, which we quantified previously as orthogonality, help to identify pathways with high and low degrees of interactions. Computationally, we find that EG exhibits a lower level of interaction compared to many natural and some synthetic pathways, which we believe makes it a more robust substrate than other alternative substrates such as formate or methanol. Hence, these interactions provided a rational basis for selecting and engineering a novel substrate-utilizing pathway in *E*. *coli*. To our knowledge, this work demonstrates the first de novo design of a bioproduction pathway from alternative substrates based on the orthogonality metric.

Our results demonstrate the applicability of *E*. *coli* to use a new and novel substrate that had not been considered previously. Initial characterization of cell growth using shake flasks showed a substrate uptake rate of approximately 5 mmol/gDW·h. At typical cell densities for industrial processes (10–100 g/L) [54], this corresponds to a net flux of 3–30 g/L h, well above the required 3–4 g/L h productivity typically needed for growth-independent production [55]. Further characterization of this strain led us to determine that there was some oxygen sensitivity, especially during early exponential phase. We believe that this is likely caused by metal catalyzed oxidation of 1,2-propanediol oxidoreductase (*fucO*) in the presence of excess aeration and could be addressed by using O_2_-tolerant Zn^2+^-dependent variants.

Based on previous reports from literature, two *fucO* mutants predicted to have improved O_2_-tolerance were tested. Ultimately, the I7L/L8V mutant (LMSE11) showed the greatest EG assimilation and growth rate/yield, and was thus used for subsequent experiments. It should be noted, however, that the growth yield with this strain was still lower than the value predicted by modeling: 0.28 g DW/g EG compared to 0.43 g DW/g EG predicted under aerobic conditions at the measured substrate uptake rate. This difference suggests that the enzyme is still not operating as efficiently as possible and that additional improvements may be achievable through enzyme engineering. In particular, directed evolution or rational design approaches could be used to improve the enzyme activity and oxygen tolerance of *fucO* [56, 57]. Such improved enzymes can lead to further improvements in glycolate production from EG.

An important observation made was a reduction in the substrate uptake rate during oxygen limiting conditions. When oxygen is limiting, reduced metabolites and electron carriers can accumulate [58]. Since the first two steps of the EG-assimilation pathway are NAD^+^-dependent, we believe that the reduced oxygen results in increased NADH pools, leading to a decrease in the rates of reaction catalyzed by *fucO* and *aldA* (which each require NAD^+^ as a cofactor). This change in the rates had a net effect of lowering the flux of EG into the cell. This finding necessitates a further study of cellular physiology under EG utilization in order to understand the trade-off in yield and productivity as a function of the dissolved oxygen feeding in bioreactors. For example, in the first set of bioreactor experiments we found that using a higher aeration rate led to a higher overall glycolate titer (4.1 g/L at 150 mL/min compared to 2.5 g/L at 50 mL/min). Comparatively, by maintaining a target respiratory quotient during the second set of bioreactor experiments, an even higher product titer was achieved at the lower aeration rate (10.4 g/L at 50 mL/min). Hence, optimization of aeration in the bioreactor would substantially improve economic performance, not only in terms of product formation but also in terms of the absolute cost of aeration.

In this study, computational modeling allowed us to predict and better understand the metabolic behaviour for EG utilization. Through flux balance analysis (FBA) it was demonstrated that EG is assimilated into native metabolism through glyoxylate oxidase, and that this enzyme could serve as an effective metabolic valve for the control of cell growth and production. FBA predicted that glycolate was unlikely to be produced under aerobic conditions, however under oxygen-limiting conditions glycolate was likely to accumulate. This agreed well with bioreactor studies, which showed that glycolate accumulation could be induced through a reduction in the aeration. Modeling also predicted a reduction in EG uptake rate, under oxygen-limiting conditions, which was equally seen experimentally.

During the experiments, we also observed small amounts of acetate and trace amounts of ethanol in the fermentation media during the micro-aerobic glycolate production phase. The presence of acetate and ethanol in the fermentation medium, typical products of anaerobic growth, suggests that EG may be a suitable feedstock for the production of other anaerobic products. Comparatively, ethanol production, depending on the ratio of the EG uptake flux and the OUR (Fig. 6b). These results, and the absence of acetate formation in the modeling predictions, highlight that while modeling is useful for obtaining a general understanding of metabolic behaviour, the model accuracy depends largely on the completeness and accuracy of the constraints used. More specifically, many regulatory changes occur in cells when they are under oxygen-limiting conditions that were not accounted in the models and which may cause some discrepancies. Hence, it is possible that taking into account other anticipated regulatory modifications can lead to more accurate predictions.

Finally, by extending the observations from FBA, we were able to correlate the glycolate production with the respiratory quotient. Thus, by measuring the respiratory quotient in real-time, using data from the process mass spectrometer, we were able to use this correlation to improve and control glycolate production during the course of the fermentation. This computational-based strategy, which led to the highest glycolate titers and production rate at a relatively low aeration rate, could be employed in other production systems to achieve similar improvements in production. Ultimately, we believe that our results successfully demonstrate the design and optimization of bioproduction pathways using computational tools and their metrics (i.e. orthogonality), particularly for the design of pathways for unconventional, non-sugar feedstocks.

## Conclusions

The results described in this study establish a framework for future production of chemicals in *E*. *coli* using EG as a substrate. We describe, for the first time, the successful production of glycolate from EG using the substrate as a feedstock for growth and for production. We also used metabolic modeling to identify the oxygenation conditions that optimize the production of glycolate and validated the strategy experimentally, thereby illustrating the value of metabolic models in bioprocess optimization. Further, we also showed that ethylene glycol utilization pathways are highly orthogonal to cellular metabolism making it an important feedstock for accelerated metabolic engineering using dynamic control. We also illustrated the value of orthogonal pathways for dynamic control of metabolism using oxygen as a control valve to switch to and optimize glycolate production. We believe this can have important implications in the future for integrating biorefineries into industries where carbon dioxide can be captured and converted from point sources.

## Methods

A summary of the investigations is presented in the Introduction (Fig. 1). First, the orthogonality was evaluated to compare EG as a feedstock for selected bioproducts (Fig. 1a). Based on this analysis, the EG to glycolate pathway was selected to be used as a case study for orthogonality-based pathway design. The production system and its anticipated metabolic behaviour was first characterized using flux balance analysis (FBA) and flux variability analysis (FVA) (Fig. 1b). Following this analysis, the pathway was tested in shake flasks, to confirm that the pathway could indeed support both cell growth and glycolate production, as predicted computationally (Fig. 1c). For this experiment, two strains were tested, each having a different *fucO* mutant, informed based on previously published studies (discussed further in text). The best growing strain was then selected for further testing in bioreactors (Fig. 1d). As per the identified metabolic valve (glycolate oxidase), two reduced air flow rates were tested in the production stage to evaluate the use of oxygen as a control method for two-stage growth and production. Using data from the shake flasks and bioreactor experiments, flux balance analysis (FBA) was subsequently performed to gain insight on the metabolic response to oxygen level and to determine the optimal air flow rate for glycolate production (Fig. 1e). Finally, a second round of bioreactor experiments was performed to test whether glycolate production could be further improved, using the insights gained through experiments and modeling (Fig. 1f). The specific methods employed in these experimental and computational analyses are discussed in detail in the sections that follow.

### Media and cultivation conditions

Cells were grown using lysogeny broth (LB) as per manufacturer’s instructions (Bioshop, Burlington, ON) for all strain construction and fermentation pre-cultures. Pre-cultures were grown in LB media in 10 mL test tube cultures overnight and transferred to fresh 250 mL shake-flaks containing 50 mL LB, 1 mM IPTG and 10 g/L EG. After 24 h, these cells were harvested by centrifugation, re-suspended in 2 mL of residual supernatant and used as inoculum for bioreactor or minimal media shake-flasks for characterization at 37 °C.

When characterizing strains (see Fig. 1c), cells were grown in M9 minimal media with the following compositions: 1.0 g/L NH_4_Cl, 3.0 g/L KH_2_PO_4_, 6.8 g/L Na_2_HPO_4_, 0.50 g/L NaCl. Supplements of yeast extract at 2 g/L were added to minimal media. EG was used as the carbon source at a concentration of ~ 10 g/L. IPTG was used at a concentration of 1 mM to induce expression of the EG assimilation pathway. A trace metal solution was prepared according to the following composition prepared in 0.1 M HCl per litre and added to the media at a concentration of 1/1000: 1.6 g FeCl_3_, 0.2 g CoCl_2_⋅6 H_2_O, 0.1 g CuCl_2_, 0.2 g ZnCl_2_⋅4H_2_O, 0.2 g NaMoO_4_, 0.05 g H_3_BO_3_. 1 M MgSO_4_ and 1 M CaCl_2_ was also added to the media at a concentration of 1/500 and 1/10,000, respectively. For all cultures, carbenicillin was added as appropriate at 100 µg/mL. All characterization experiments were conducted with 50 mL media in 250 mL shake flasks, continuously agitated at 230 rpm and at 37 °C. Culturing techniques employed in the bioreactors are described below.

### Culturing techniques in reactors

Applikon MiniBio500 500 mL fermentation vessels with a 300 mL working volume were used for cultivating strains in bioreactors. The fermentation vessels were equipped with condensers to prevent changes in volume due to aeration. Dissolved oxygen and pH probes were used in accordance with the manufacturers operating guidelines. pH was maintained at 7 with the addition of 3 N KOH. Growth conditions were maintained at 37 °C. Bioreactors were inoculated with pre-culture (previously described) at OD ~ 0.4 (approx. 0.23 gDW/L). In total, four bioreactor cultivations were conducted using *E. coli* strain LMSE11 in minimal media, supplemented with yeast extract at 2 g/L. The bioreactors contained 1 mM IPTG to maintain induced expression of the EG pathway genes. All bioreactor cultivations were carried out in fed-batch. Systematic changes in aeration rate and impeller speed were applied between cultivations, as detailed below. Flowrate was controlled using a Books Instruments mass flow controller (GF Series) and gas was analyzed using Thermo Scientific™ Sentinel dB mass spectrometer for online gas measurement.

Cultivations 1 and 2: Cells were grown at an impeller speed of 1000 rpm and sparged with air to maintain oxygen at 300 mL/min (1 v/vm). At 20 h, the aeration was reduced to 150 mL/min (0.5 v/vm) or 50 mL/min (0.16 v/vm) to simulate high and low secondary aeration rates, and the impeller speed was dropped to 500 rpm. See Fig. 1d.

Cultivation 3: Cells were grown at an impeller speed of 1000 rpm and sparged with air to maintain oxygen at 600 mL/min (2 v/vm) during the growth phase. In the production phase, the impeller speed was reduced to 500 rpm while the aeration rate was dropped to 100 mL/min (0.33 v/vm). See Fig. 1f.

Cultivation 4: During the growth phase, cells were sparged with air to maintain oxygen at 50 mL/min (0.16 v/vm) throughout the cultivation and the impeller speed was controlled to maintain a minimum oxygen level of 20%, up to a maximum impeller speed of 1000 rpm. In the production phase, the impeller speed was reduced as necessary to achieve a respiratory quotient (RQ) of ~ 0.4. See Fig. 1f.

### Analytical methods

Analysis of fermentation production was measured via high performance liquid chromatography (HPLC). We used a Bio-rad HPX-87H organic acids column with 5 mM H_2_SO_4_ as the eluent and a flowrate of 0.4 mL/min at 50 °C. Organic acids were detected at 210 nm. Cell densities of the cultures were determined by measuring optical density at 600 nm (GENESYS 20 Visible Spectrophotometer). Cell density samples were diluted as necessary, to fall within the linear range. A differential refractive index detector (Agilent, Santa Clara, CA) was used for analyte detection and quantification. Yields were calculated between two time points, whereas the cumulative yield was calculated between the initial and final measurements.

### Plasmids and strains

Genes *fucO* and *aldA* were cloned from *E. coli* MG1655 genomic DNA and assembled using Gibson Assembly [59] into a pTrc99a vector. RBS sequences were placed onto the overhang of the forward primer. AACAAAATGAGGAGGTACTGAG was the RBS sequence used in front of *aldA*. AAGTTAAGAGGCAAGA was the RBS sequence used in front of *fucO*. The *trc* promoter was used to drive expression. Wild-type strains of *E*. *coli* MG1655 were obtained from the Coli Genetic Stock Centre (Yale).

### Flux balance analysis

Flux balance analysis (FBA) was performed using both MATLAB R2015a installed with COBRA 2.0 toolbox and the COBRApy toolbox with Python 3.7.3, using the GLPK linear solver (GNU Project). All modeling was performed using either the genome scale model iAF1260 or the *E. coli* core model, as indicated below. The ATP maintenance reaction was left unchanged for both models, corresponding to a value of 8.39 mmol/gDW·h. Both the core and genome scale models were modified by the addition of missing reactions in the EG assimilation pathway, including the transport and exchange of EG by free diffusion (no proton translocation). The default glycolate transport and exchange reactions were used for the genome scale model and equivalent reactions were added for the core model. Formate exchange was knocked out for all analyses in which oxygen was present.

The orthogonality evaluation was performed using the core model, as previously described [4]. All other FBA and FVA analyses were performed using the iAF1260 genome scale model, with biomass set as the objective function. For the general FBA and FVA analyses (Fig. 1b), EG uptake was constrained to 10 mmol/gDW·h, while oxygen uptake was constrained to 20 mmol/gDW·h for aerobic conditions and 5 mmol/gDW·h for micro-aerobic conditions. For the FVA analysis, the fraction of optimum value was set to 0.5. When evaluating oxygen effect (Fig. 1e), EG uptake was constrained to 5 mmol/gDW·h, as measured during the early exponential growth phase in the shake flask experiments. For this evaluation, oxygen flux was varied from 3.5 up to its maximum value of 6.6 mmol/gDW·h.

### Orthogonality

Orthogonality is a metric that quantifies the interconnectedness between a production pathway and a microorganism’s native growth pathways [4, 6]. Here the orthogonality of selected substrate-product pairs was evaluated using the same approach we previously described [4]. Any metabolic network can be represented as a stoichiometric solution space, for which subspaces can be defined. All reactions contributing to chemical production compose the product subspace (S_t_), while all those contributing to biomass production compose the biomass subspace (S_x_). To determine the orthogonality of the given chemical product and metabolic network, the elementary flux modes (EFMs) must first be determined and assigned to the product and biomass subspaces. All EFMs with non-zero chemical flux and zero biomass flux are assigned to the product subspace, while those with non-zero biomass flux and zero chemical flux are assigned to the biomass subspace. The degree of similarity between these two subspaces is defined as the average similarity (AS) coefficient, and is determined by the number of shared reactions, normalized by the size of the supporting biomass mode (Eq. 1). The orthogonality, which evaluates the degree of separation, is then determined using the AS (Eq. 2). See Additional file 1: Figure S1.

1 $$\overline{AS} = \frac{{\mathop \sum \nolimits_{i = 1}^{m} \mathop \sum \nolimits_{j = 1}^{n} \frac{{e_{i}^{t} \cdot e_{j}^{x} }}{{e_{j}^{x} \cdot e_{j}^{x} }}}}{mn}$$

2 $$OS = 1 - \overline{AS}$$

## Supplementary Information

**Additional file 1: Figure S1.** The effect of microaerobic (oxygen-limiting) conditions on glycolate production, as predicted by modeling at different substrate uptake rates (SUR) and non-growth associated ATP maintenance (NGA ATPM) values. (A) SUR of 5 mmol/gDW·h and default NGA ATPM value (8.39 mmol/gDW·h.). (B) SUR of 0.7 mmol/gDW·h and NGA ATPM value of 4.8 mmol/gDW·h. (C) SUR of 0.7 mmol/gDW·h and NGA ATPM value of 3.2 mmol/gDW·h. (D) SUR of 0.7 mmol/gDW·h and NGA ATPM value of 1.6 mmol/gDW·h. In each case, flux balance analysis (FBA) was used to predict the glycolate yield (mol glycolate/mol EG), cell growth rate (gDW/gDW·h), respiratory quotient (RQ, mol CO2/mol O2) and the substrate specific productivity (SSP, g glycolate/g EG·h) over a range of oxygen uptake rates (OURs, mmol O2/gDW·h). All values other than the SSP are shown on the primary vertical axis. An RQ value of 0.4 (shown by the dotted black line) was selected for the RQ-based strategy employed in the final bioreactor experiment. The molar yield (mol glycolate/mol EG) predicted at this RQ value is indicated for each case.

## Data Availability

We will provide our data on modeling through git hub upon publication.

## Abbreviations

CO_2_: Carbon dioxide
EG: Ethylene glycol
TCA: Tricarboxylic acid
FBA: Flux balance analysis
DHAP: Dihydroxyacetone phosphate
3PG: 3-Phosphoglycerate
2PG: 2-Phosphoglycerate
PEP: Phosphoenolpyruvate
AcCoA: Acetyl-CoA
Cit: Citrate
Icit: Isocitrate
Akg: Alpha-ketoglutarate
SucCoA: Succinyl-CoA
Succ: Succinate
Fum: Fumarate
Mal: Malate
Oaa: Oxaloacetate
TSA: Tartronate semialdehyde
FVA: Flux variability analysis
PDO: 1,2-Propanediol oxidoreductase
GCALDD: Glycolaldehyde dehydrogenase
GLYCTO2: Glycolate oxidase
GLXCL: Glycolate carboligase
TRSARr: Tartronate semialdehyde reductase
GLYCK2: Glycerate kinase
PGM: Phosphoglycerate mutase
ENO: Enolase
PYK: Pyruvate kinase
MALS: Malate synthase
ME1/ME2: Malic enzyme
PDH: Pyruvate dehydrogenase
PFL: Pyruvate formate lyase
CS: Citrate synthase
MCO: Metal-catalyzed oxidation
HPLC: High-performance liquid chromatography
RQ: Respiratory quotient
SSP: Substrate specific productivity
OUR: Oxygen uptake rate
