# Supplementary material for: Engineering Escherichia coli for the utilization of ethylene glycol
Source: Microb Cell Fact. 2021 Jan 22;20:22. doi: 10.1186/s12934-021-01509-2 (PMC7821661; doi:10.1186/s12934-021-01509-2)
Supplement: Supplementary file 1 — Additional file 1: Figure S1. The effect of microaerobic (oxygen-limiting) conditions on glycolate production, as predicted by modeling at different substrate uptake rates (SUR) and non-growth associated ATP maintenance (NGA ATPM) values. (A) SUR of 5 mmol/gDW·h and default NGA ATPM value (8.39 mmol/gDW·h.). (B) SUR of 0.7 mmol/gDW·h and NGA ATPM value of 4.8 mmol/gDW·h. (C) SUR of 0.7 mmol/gDW·h and NGA ATPM value of 3.2 mmol/gDW·h. (D) SUR of 0.7 mmol/gDW·h and NGA ATPM value of 1.6 mmol/gDW·h. In each case, flux balance analysis (FBA) was used to predict the glycolate yield (mol glycolate/mol EG), cell growth rate (gDW/gDW·h), respiratory quotient (RQ, mol CO2/mol O2) and the substrate specific productivity (SSP, g glycolate/g EG·h) over a range of oxygen uptake rates (OURs, mmol O2/gDW·h). All values other than the SSP are shown on the primary vertical axis. An RQ value of 0.4 (shown by the dotted black line) was selected for the RQ-based strategy employed in the final bioreactor experiment. The molar yield (mol glycolate/mol EG) predicted at this RQ value is indicated for each case. [file 12934_2021_1509_MOESM1_ESM.docx]

# Supplementary Information

The OUR analysis previously presented (Figure 6) was performed using the substrate uptake rate measured during the shake flask characterization (5 mmol EG/gDW·h; see Figure 4). This analysis was used to select an RQ value that would best support glycolate production in two-stage bioreactors. Testing this strategy *in vivo* resulted in the highest glycolate yield and titer of all four bioreactor experiments (0.8 g/g and 10.4 g/L, respectively. It was observed, however, that the substrate uptake rate (SUR) was significantly reduced during the experimental production stage; 0.7 mmol/gDW·h compared to the value of 5 mmol/gDW·h initially assumed. As the substrate uptake rate is expected to have a measurable effect on the modeling predictions, the OUR analysis was repeated at the lower substrate uptake rate. Upon testing a substrate uptake rate of 0.7 mmol/gDW·h, the FBA solution became infeasible as the rate of substrate provision was not high enough to meet the non-growth associated ATP maintenance (NGA ATPM) requirements set by the model (8.39 mmol ATP/gDW·h).

It has been shown experimentally, that the ATP maintenance requirements of *E. coli* can vary under different conditions, and in particular, the NGA ATPM is expected to decrease under conditions of carbon starvation (1). Arguably, we stipulate that oxygen starvation could also lead to similar reductions in the NGA ATPM. Further, since the iAF1260 genome scale model was first published, more recent studies have also reported lower NGA ATPM values for *E. coli* under fed conditions, such as the value of 3.2 mmol ATP/gDW·h reported by Taymaz-Nikerel and colleagues (2). In light of these considerations, the OUR analysis was repeated at the reduced substrate uptake rate of 0.7 mmol/gDW·h, at three lower NGA ATPM values (3.2 mmol ATP/gDW·h ± 50%). At these lower NGA ATPM flux values, the solution is feasible and the results from this analysis are shown in Supplementary Figure 1B-D below. For comparison, the non-normalized results for the analysis performed in Figure 6 (SUR of 5 mmol/gDW·h and NGA ATPM of 8.39 mmol/gDW·h) were also included (Supplementary Figure 1A). It should be noted that in cases where the NGA ATPM was reduced, the growth-associated ATP maintenance remained unchanged. This is not expected to significantly change the results as the growth rate was near-zero in all three cases.

In order to compare the model predictions for all four SUR and NGA ATPM scenarios, the yields predicted by each at an RQ of 0.4 were determined and are shown in Supplementary Figure 1. Unlike at the higher SUR of 5 mmol/gDW·h, the growth rates predicted at an SUR of 0.7 mmol/gDW·h are consistently near zero (Supplementary Figure 1B-D), as was observed experimentally during the corresponding bioreactor production phase (Figure 7B). Of the three reduced NGA ATPM values tested, the glycolate yield predicted at a NGA ATPM of 3.2 mmol/gDW·h is most closely aligned with the yield that was observed experimentally (0.65 mol/mol predicted compared to 0.66 mol/mol measured). While we also cannot conclusively say that the NGA ATPM was exactly 3.2 mmol/gDWh, the results do suggest that the cells were likely operating at a NGA ATPM lower than the value initially assumed in the model (8.39 mmol/gDW·h). As a consequence, these results also suggest that the oxygen uptake rate (OUR) was lower than initially predicted for an RQ of 0.4 and the higher SUR. These results demonstrate the importance of refining models as more experimental data becomes available.

1. Biselli E, Schink SJ, Gerland U. Slower growth of Escherichia coli leads to longer survival in carbon starvation due to a decrease in the maintenance rate . Mol Syst Biol. 2020;16(6):1–13.
2. Taymaz-Nikerel H, Borujeni AE, Verheijen PJT, Heijnen JJ, van Gulik WM. Genome-derived minimal metabolic models for Escherichia coli MG1655 with estimated in vivo respiratory ATP stoichiometry. Biotechnol Bioeng. 2010;107(2).

**
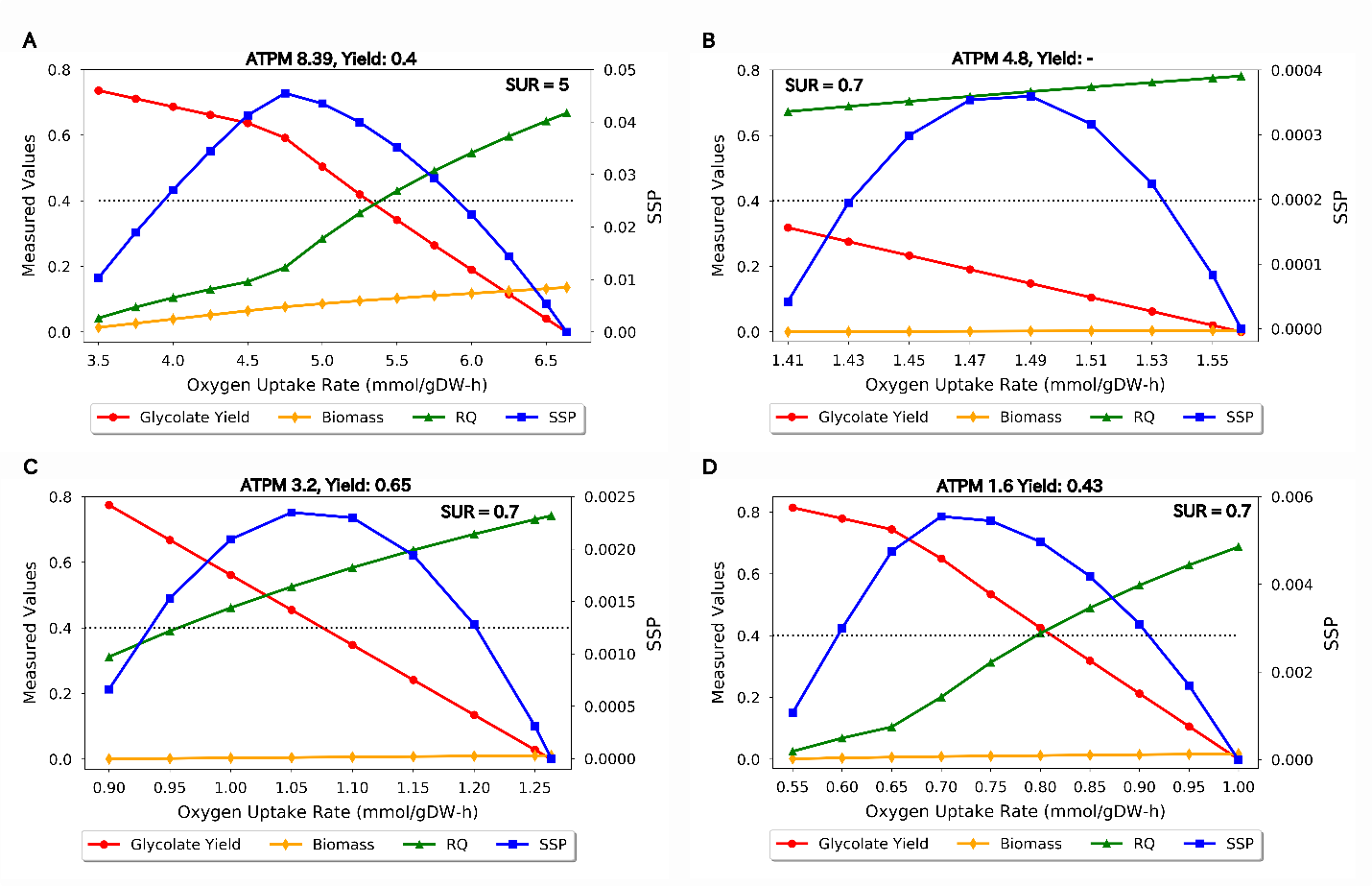
Supplementary Figure 1.** The effect of microaerobic (oxygen-limiting) conditions on glycolate production, as predicted by modeling at different substrate uptake rates (SUR) and non-growth associated ATP maintenance (NGA ATPM) values. (**A**) SUR of 5 mmol/gDW·h and default NGA ATPM value (8.39 mmol/gDW·h.). (**B**) SUR of 0.7 mmol/gDW·h and NGA ATPM value of 4.8 mmol/gDW·h. (**C**) SUR of 0.7 mmol/gDW·h and NGA ATPM value of 3.2 mmol/gDW·h. (**D**) SUR of 0.7 mmol/gDW·h and NGA ATPM value of 1.6 mmol/gDW·h. In each case, flux balance analysis (FBA) was used to predict the glycolate yield (mol glycolate/mol EG), cell growth rate (gDW/gDW·h), respiratory quotient (RQ, mol CO_2_/mol O_2_) and the substrate specific productivity (SSP, g glycolate/g EG·h) over a range of oxygen uptake rates (OURs, mmol O2/gDW·h). All values other than the SSP are shown on the primary vertical axis. An RQ value of 0.4 (shown by the dotted black line) was selected for the RQ-based strategy employed in the final bioreactor experiment. The molar yield (mol glycolate/mol EG) predicted at this RQ value is indicated for each case.
